# Introduction to LJPC issue 9.4. July/August 2017

**DOI:** 10.1080/17571472.2017.1347140

**Published:** 2017-07-17

**Authors:** Paul Thomas

This Issue of LJPC shows how writing, painting, the arts and stories can reveal different, complementary aspects of health. Together they can build up rich pictures that are invisible to any one expression and different from the insights gained from facts and statistics about diseases. They can do more than reveal aspects of health; they can improve it.

Claire Brash interviewed Stephen Bergman, Professor of medical humanities at New York University. He wrote the novel ‘The House of God’ under the name Samuel Shem. This book sold over 3 million copies, and exposed the connection between values, good relationships and healing. ‘What led you to write the House of God?’ she asked. ‘Outrage’ he answered. ‘What made you undertake this interview, Claire?’ Her reply: ‘Around 80% of medical students with mental health issues feel under-supported’. That sounds like a good reason to highlight non-medical aspects of health.

Malcolm Torry makes the case for a ‘spiritual assessment’ to be undertaken to help understand a patient as a whole person. Different belief systems lead to different expectations that lead to different interpretations of what health means to them. Primary care practitioners must be aware of these nuances and handle them sensitively.

Michael Druquer describes ways that the novels, paintings and music can bear witness to the human condition and help to see dynamic, social aspects of health, beyond (and as well as) their diseases.

Francesco Carelli describes how the paintings of Francis Bacon from the 1940s ‘bore witness to the shattered psychology of the time’, another example of how we can see different aspects of health through the arts than through statistics of diagnostic categories.

Finally, we say goodbye to Elizabeth June Horder. She is well known as the wife of John Horder, one of the greatest GPs of all time. But she was her own person. A force to be reckoned with. She was herself a pioneer GP and beautiful in every way. Take the time to read her account of general practice before and after the NHS began. Search the LJPC website with the name ‘Horder’ and you will see her paper ‘Recollections of general practice’; read this and then read John’s autobiography to see those days in colour, beyond the statistics. As well as reminding us that great people attract great people, Elizabeth and John reveal the origins of the principles of quality general practice that we still value today. Quality general practice improves the health of families and communities as well as treating the diseases of individuals. They explain why generalism really matters, in health care and in other public services. We must remember this in these dangerous days when faced by those who want to turn our precious NHS into a machine where specialists and super-specialists help us to see diseases more and more, and people less and less.

